# Organic Food in Athletes Diet—Narrative Review of Alternative Products in Sports Nutrition

**DOI:** 10.3390/nu16142347

**Published:** 2024-07-20

**Authors:** Hubert Dobrowolski, Klaudia Kopczyńska, Renata Kazimierczak, Ewa Rembiałkowska, Dariusz Włodarek

**Affiliations:** 1 School of Medical & Health Sciences, University of Economics and Human Sciences in Warsaw, Okopowa 59 Str., 01-043 Warsaw, Poland; 2 Department of Functional and Organic Food, Institute of Human Nutrition Sciences, Warsaw University of Life Sciences (SGGW), Nowoursynowska 159c Str., 02-776 Warsaw, Poland; klaudia_kopczynska@sggw.edu.pl (K.K.); renata_kazimierczak@sggw.edu.pl (R.K.); maria_rembialkowska@sggw.edu.pl (E.R.); 3 Department of Dietetics, Institute of Human Nutrition Sciences, Warsaw University of Life Sciences (SGGW), Nowoursynowska 159c Str., 02-776 Warsaw, Poland; dariusz_wlodarek@sggw.edu.pl

**Keywords:** sports diet, antioxidants, sports food, substitutes for athletes, calcium, vitamin D, iron, proteins, PUFA

## Abstract

Athletes are characterized by special nutritional needs. Meeting their dietary needs associated with intensive exercise is a prerequisite for effective training and success in sports competitions. Hence, it is important to supply key performance nutrients, such as macronutrients, antioxidants, calcium, vitamin D, or iron, in adequate quantities. The increased need for these nutrients makes it necessary to look for food products on the market that more fully cover these needs. Such products may include organic foods. According to research, they have unique properties and are richer in selected nutrients, such as antioxidants. Hence, the aim of this review was to analyze the available literature as to whether organic foods have the potential to more fully cover the increased nutritional requirements of athletes for selected nutrients compared to conventionally produced foods. A narrative review of current literature was carried out. As the analysis showed, organic foods are characterized by a higher content of antioxidant bioactive compounds, a higher content of *n*-3 fatty acids, a better n:3/n:6 ratio, and a more optimal amino acid composition, which may result in an appropriate dietary ration design for athletes. In conclusion, organic food appears to be an interesting alternative to meet the special nutritional needs of professional and amateur athletes.

## 1. Introduction

Athletes are characterized by specific nutritional needs. Exercise stresses many metabolic pathways in which micronutrients are required, and training can result in biochemical adaptations in muscles that increase the need for certain micronutrients, such as calcium, vitamin D, iron, and some antioxidants [1]. Also, macronutrient requirements are altered compared to the general population and adjusted to the characteristics of exercise [2,3,4]. Many nutritional studies conducted in the athlete population have shown insufficient intake of selected nutrients, which may be reflected in reduced physical performance. There is, therefore, a justified need to increase the intake of nutrient-rich products, which will provide a valuable source of nutrients to cover the increased needs of athletes.

Organic foods are agricultural crops and processed products derived from organic farming and processing, which are certified after the mode of production has been inspected for compliance with applicable laws by certification bodies appointed for this purpose and labeled accordingly. Organic farming aims to provide food products of high quality, while preserving the welfare of the plant environment and livestock [5]. The main objective, but also the challenge of organic farming, is to balance the costs of agricultural production and ensure the highest possible quality of crops and livestock [5]. The rules of organic food production in the EU are governed by Regulation (EU) 2018/848 of the European Parliament and the Council of 30 May 2018 on organic production and labeling of organic products [5].

Certified organic food is characterized by an almost complete absence of synthetic pesticide residues and antibiotics, and a complete absence of synthetic food additives—colorings, preservatives, flavor enhancers, and others—which are prohibited in organic food production. Genetically modified organisms (GMOs) or their derivatives cannot be used in any form either [6,7]. Traditional methods of processing and preservation of products are preferred, based on minimal interference, allowing to maintain high-quality values of organic products [5,8]. Organic products, compared to their conventional analogues, contain more certain bioactive components [9,10], selected fatty acids [11,12], or selected vitamins and minerals [13,14]. The health benefits and positive health effects of eating organic products are the most frequently cited motivators for choosing this type of food [15,16].

Athletes have special nutritional needs, and there are still problems providing adequately valuable products—organic products are these kinds of foods. The purpose of this review was to provide an in-depth analysis of the possibility of using organic products as a better alternative to conventional products in the diets of athletes.

## 2. Search Strategy and Selection Criteria

To accomplish this work, a careful and cautious selection of papers was undertaken in order to include only those of the highest quality in the review. PubMed, Scopus, and Google Scholar databases were used during the literature analysis. Original research articles, review papers, and meta-analyses were included in the review. When searching the databases, combinations of keywords, such as “athletes”, “sportsmen”, “sportswoman”, “sportspeople”, “physical performance”, “physical fitness”, and “physical activity” were used for studies on athletes, or “organic food”, “ecological food”, “organic vs. conventional”, and “bioavailability” for studies on organic food, combined with various combinations of the words “dietary”, “nutritional”, “intake”, “recommendations”, “antioxidants”, “vitamin C”, “vitamin E”, “vitamin D”, “calcium”, “iron”, “proteins”, “amino acids”, “EAA”, “BCAA”, “polyunsaturated fatty acid”, “PUFA”, “essential fatty acids”, “*n*-3 fatty acids”, “omega-3 fatty acids”, “CLA”, “caffeine”, “ergogenic”, “caffeine”, “carnosine”, “alanine”, “pesticides”, “contamination”, and “exposure”. Before inclusion in the article, identified studies were reviewed for subject matter and quality based on the text of the entire articles. In addition, from the identified papers included in the review, the reference lists were screened for inclusion of possible further papers. By design, the literature review was based on papers published during the last decade, but also included highly regarded older publications.

## 3. Organic Food and Antioxidant Potential

Organic plant products are particularly valuable as a source of selective antioxidants, such as polyphenols (including phenolic acids and flavonoids), compared to their counterparts from conventional production [10]. Moreover, organic products, such as milk and meat, are characterized by a high content of carotenoids and vitamin E isomers [11,12]. As studies indicate, chronic training induces continuous oxidative stress in the cells due to the fact that athletes consume more oxygen [17]. Dietary intake of antioxidants by athletes is crucial. However, there is no conclusive evidence that antioxidant supplementation supports physical performance. The Academy of Nutrition and Dietetics (AND), Dietitians of Canada (DC), and American College of Sports Medicine (ACSM), in their position statements, recommend consuming well-chosen foods rich in antioxidants to prevent deficiencies [1]. Similar observations were pointed out in a literature review by Antonioni et al. (2019), where the authors emphasized that athletes’ antioxidant needs are the same or close to the Recommended Dietary Allowance (RDA) and can be provided with a well-balanced diet, and antioxidant supplementation alone may be required during intense training periods [18]. It appears that over-supplementation with antioxidants often has an opposite result to the expected one, and negatively affects the training process [1,18] and increases oxidative stress during training. Therefore, it is important to ensure the provision of an adequate dose of antioxidants with a properly put together food ration, containing products that are rich sources of bioactive compounds.

Many existing studies have looked at comparing the antioxidant content in organic and conventional products. The meta-analysis by Baranski et al. (2014) is the most comprehensive to date. Based on 343 studies from around the world, they found significantly higher levels of polyphenols in plant materials from organic production than conventional production. There were differences ranging from 19% for phenolic acids through to 50% for flavonols and anthocyanins, to as high as 69% for flavanones [10]. Since then, many new studies have been performed, most of which confirmed the results obtained in the meta-analysis described above. Organic vegetables contained more tocopherols (butter squash), total carotenoids, and flavonoids (bell peppers) compared to conventional ones. Organic fruits contained more phenolic acids, flavonoids (blackcurrant and apples), as well as vitamin C and total anthocyanins (black currants) [19,20,21,22,23,24,25]. Table 1 shows studies presenting a comparison of antioxidant content depending on the agricultural system (organic vs. conventional). Recently published reviews showed a similar trend of organic food in terms of the content of selected bioactive compounds, minerals, and dry matter [13,26]. However, not all studies provided such optimistic results. The opposite observation was made in a study by Dutra et al. (2018), where the authors, comparing Brazilian organic and conventional grape juices and wines, found no significant differences in phenolic compounds and antioxidant activity, while anthocyanin content was higher in conventional products [27]. Pedro et al. (2022) also showed higher levels of total polyphenols, vitamin C, and antioxidant potential in goji berries from organic production than from conventional production. However, these differences were not statistically significant [25]. It should be noted, however, that Brazilian standards for organic food differ from European standards, which may present some obstacles when comparing products from member and non-member countries. Also, Skupień et al. (2011) did not show an advantage for any of the raspberry-growing methods. As the authors pointed out, this could be due to differences in location and weather conditions during cultivation [28].

Studies indicate that antioxidant intake among athletes is mostly at insufficient levels. A study of 118 French, highly trained athletes found that the daily supplies of vitamins E and C were too low in most subjects (81% and 60%, respectively), while more than 40% of athletes consumed too little β-carotene [33]. Also, a study involving marathon runners found an under-supply of vitamin E in almost all subjects and an under-supply of vitamin C, β-carotene, and retinol in almost one-third of subjects [34]. Similarly, a study involving volleyball players of both sexes found insufficient intake of vitamins A and E. Vitamin C intake, on the other hand, was within the recommended values for both the athletes and the control group [35]. A study involving aquatic athletes (swimming and water polo) found that 71% of men and 93% of women did not meet their needs for one or more antioxidant vitamins. The authors of the study emphasized that the low supply of antioxidants in the study group was due to a low supply of fruits and vegetables [36]. More optimistic results were obtained in a study by Devrim-Lanpir et al. (2020), where ultra-marathoners mostly met their needs or even consumed excessive amounts of micronutrients, but some of them showed an insufficient intake of vitamins A and E. However, as the authors pointed out, standards are set for the needs of the non-athlete population, and athletes themselves may have higher needs than the general population [37]. Also, a study by Dobrowolski and Wlodarek (2019), with female soccer players, showed a low intake of antioxidants by a large percentage of participants in the study [38]. A recent study by Leonhardt et al. (2024) also found an under-supply of antioxidants among World Masters Athletics Championships athletes. However, this study was based on a single 24 h interview and may, therefore, carry a curved view of the dietary content of selected nutrients [39].

Oxidative stress has been linked to a number of factors affecting physical performance, such as fatigue, muscle damage, and reduced immune function [40]. Antioxidants, including non-enzymatic ones, such as vitamins, are involved in controlling excessive oxidative stress and reducing the risk of adverse effects in professional athletes [41]. However, as the research indicates, the combined action of multiple compounds derived from a diet rich in fruits and vegetables cannot be replaced by supplementation with a single or a combination of antioxidants [33], which only highlights how important a role antioxidant-rich organic fruits and vegetables can play. A diet rich in antioxidants may really be a nonpharmacologic and natural opportunity to maintain a physiological antioxidant status in sportspeople [41].

## 4. Iron

Iron remains one of the key micronutrients for athletes—it is responsible for oxygen transport and energy production [42], as well as its cellular utilization is crucial for endurance performance [43]. Athletes are at particular risk of developing iron deficiency. This is because they experience more frequent iron loss, such as from gastrointestinal bleeding, exercise-induced hematuria, or loss of iron with sweat during training [44]. Women involved in professional sports are particularly vulnerable to iron deficiency, due to iron loss not only from the factors mentioned above, but also from the menstrual cycle, among others [44]. As highlighted in the AND, ACSM, and DC position paper, iron requirements for all female athletes may be increased by up to 70% of the estimated average requirement [1]. This position seems to be particularly relevant to young athletes, as studies have shown a high percentage of individuals with poor iron status among young athletes, despite the fact that their iron intake was in line with recommended values [45].

Organic foods have also been studied for their iron content. In a meta-analysis in 2016, Średnicka-Tober et al. showed that the iron content of organic milk was up to 20% higher compared to conventional milk [11]. Different results were obtained by Qin et al. (2021), showing that conventional milk contained more iron than organic milk [46]. However, it is worth noting for both of these studies that milk is not a good source of iron in the diets of adults, and it is difficult to expect that switching from conventional to organic milk could significantly affect its supply in the diet. As research on milk consumption patterns indicates, children and adolescents consume significantly more milk compared to adults, with men consuming more than women [47]. Ozen (2015), on the other hand, indicated in a systematic review that plain water is the main source of fluids, with milk consumption being higher among children [48]. Additionally, studies have indicated a reduction in milk consumption in favor of sugar-sweetened beverages [49]. As milk is itself poor in iron and its consumption decreases with age, a reasonable assumption is that it will not be a good source in the diet of adult athletes. Plant products might be an important source of iron. Shaw et al. (2022), analyzing the benefits of vegan diets among athletes, reported that beans, peas, lentils, edamame, chickpeas, nuts, seeds, whole grains, fortified bread, and cereals are important sources of iron in athletes’ diets [50]. As Worthington (2001) pointed out in analyzing a study of the nutrient content of organic products (fruits, vegetables, and grains), their average iron content was more than 20% higher, compared to conventional products [14]. Also, Hunter et al. (2011) found, based on 77 different scientific studies, 3.3% more iron in organic crops compared to conventional ones—but the differences were not statistically significant [51]. In the paper by Giampieri et al. (2022), the data were divergent, with some studies showing more iron in organic products and other studies showing no differences [26]. However, these studies should be analyzed with caution. Higher iron content does not necessarily mean its higher bioavailability, which may vary depending on the cultivation method. In a study by Drakou et al. (2014), organic lentils exhibited a lower predicted iron bioavailability than conventional lentils [52]. Findings on the usefulness of organic plant products as a source of iron are, therefore, inconclusive. In animal products, on the other hand, studying beef, Miotello et al. (2016) observed significantly higher iron contents in organic meat, compared to conventionally farmed meat [53]. Different results were obtained by Karwowska and Dolatowski (2013), where in a study on pork meat, they reported no statistical differences in iron content and iron loss due to storage when comparing conventional and organic foods [54].

It is worth noting, however, that despite the often-contradictory results obtained in studies, all, to our knowledge, have shown higher iron content in organic products, or no statistically significant difference between conventionally and organically produced foods. Thus, it can be assumed that consumption of organic foods may be associated with higher iron intake. However, the nutritional status of iron should be monitored for possible deficiencies, especially in the case of female athletes, who, as mentioned earlier, are particularly vulnerable. As studies indicate, athletes often consume iron in amounts comparable to the recommended values, with results varying depending on the study methodology adopted and the study group. Table 2 presents the dietary iron supply of athletes according to studies from recent years. At the same time, it should be noted that poor nutritional iron status can also occur in athletes with an intake close to the recommended amounts of this mineral.

## 5. Calcium and Vitamin D

Other components important to athletes’ nutrition and physical performance are calcium and vitamin D. Calcium takes part in muscle contractions, nerve transmission, blood clotting, protein utilization, and cellular communication [55]. Despite the huge role attributed to this component in maintaining health and the possibility of losing calcium with sweat, athletes do not have an increased need for calcium. However, it is recommended that they supplement with a diet containing calcium-rich products [56]. For athletes who do not meet the calcium intake requirements, supplementation (including vitamin D, if necessary) should be considered, but the goal should be to meet the calcium requirements with a well-balanced diet, with additionally adequate intake of phosphorus, protein, magnesium, and vitamins A and K [57]. Vitamin D, in turn, regulates the absorption of calcium and phosphorus and plays a key role in maintaining bone health [58], as well as being a gene modulator responsible for the process of cell growth, immune function, or protein synthesis [57]. Focusing on athletes, however, maintaining adequate serum vitamin D levels can prevent injuries and fractures, and preventing deficiencies can affect athletic performance [59]. However, it should be noted that the relationship between vitamin D and physical performance is still inconclusive [59,60,61], but vitamin D supplementation in deficient individuals has actually had an effect in improving physical performance [62].

Several studies have compared the vitamin D and calcium contents of organic products. Analysis of the vitamin D content of conventional and organic milk showed significantly higher vitamin D content in organic milk compared to conventional [63]. A different observation was made by Jakobsen and Saxholt (2009), who compared the vitamin D content of conventional and organic milk year-round and showed no statistically significant differences [64]. However, this was due to a significant disparity between the vitamin D content of milk during the open-grazing period (spring and summer) compared to the barn period (autumn and winter). Significantly higher vitamin D content was found in one of the measurement periods (May–July) in organic milk. This has to do with the fact that in spring and summer, cows in organic farms are grazed on open pastures, which affects endogenous vitamin D synthesis, while during other periods, cows’ exposure to the sun is limited, and the synthesis of this vitamin decreases. Cows from the conventional system, on the other hand, receive vitamin D supplements during the winter, which is not allowed in the organic system. Hence, the higher vitamin D content of cows raised using the conventional method during other periods (except May–July) is very likely. Analyzing the mineral content of milk, Qin et al. (2021) showed higher calcium, potassium, and phosphorus content in organic milk, compared to conventional milk [46]. Also, other studies showed a higher calcium concentration in organic vs. conventional milk [65,66]. However, there are studies showing higher calcium content in conventional milk [67]. Comparing the vitamin D content of eggs, Shwan et al. (2017) found higher vitamin D content in organic eggs compared to conventionally farmed eggs [68]. It should be noted, however, that the study was conducted in Iraq, and eggs laid by so-called free-range hens, not confined to poultry farms, were taken as organic eggs. The data should, therefore, be analyzed with some caution. Similar results, also on free-range hens, were obtained in another study [69]. On the other hand, studies on wheat showed no statistically significant differences between organic and conventional wheat [70]. However, it is worth noting that while wheat flour per 100 g of product contains significant amounts of calcium, the most commonly consumed wheat products (bread and pasta) are no longer such a good source of this mineral [71] due to the reduction in nutritional value as a result of product processing. Higher calcium contents were determined in organically grown beans compared to conventionally grown ones [72]. Also, organic eggplants contained significantly more calcium than conventional eggplants [73]. However, Hunter et al. (2011), in their review based on a number of different studies [42], showed only slightly higher levels of calcium in plant foods from organic production than from conventional ones (0.6%). Thus, it is difficult to expect a higher calcium supply from organic diets in athletes (or other consumer groups).

As with iron, there are reports on calcium and vitamin D that indicate both higher and lower contents of these components in conventionally and organically grown plant and animal products. These discrepancies were also well described in a recent study by Rahman et al. (2024) [74]. The discrepancies in the content of these components are due to the multiple factors described earlier and, in addition, to differences in the inputs used in organic farming. This is particularly evident in the case of animal feed, which in conventional agriculture can be enriched with selected components (e.g., calcium), while organic farming excludes such treatments. However, it should be pointed out that there are indications that some organic products may be richer in calcium. Unfortunately, as in the case of iron, there are insufficient studies on the bioavailability of these ingredients and the comparison of this value in organic and conventional products. It is, therefore, difficult to predict whether increased consumption of organic products would result in an improved calcium and vitamin D nutritional status in humans.

Athletes very often consume insufficient amounts of calcium and almost always consume insufficient amounts of vitamin D compared to the recommended values. Table 2 shows the intake of calcium and vitamin D in different groups of athletes in recent years. Thus, the intake of products rich in calcium and vitamin D is important from a health and performance perspective in athletes.

**Table 2:** Iron, calcium, and vitamin D content of athletes’ diets.

Study	Study Group	Method	Iron		Calcium		Vit. D		References
			mg	% Below	mg	% Below	µg/UI	% Below	
Baranauskas et al. (2020)	n = 323 High-performance athletes	24 h dietary food recall	n/d	n/d	1254.0 ± 580.2 * 1113.9 ± 501.9 *	n/d	n/d	n/d	[75]
Jenner et al. (2018)	n = 46 Male Australian football players	7-day food diary	n/d	n/d	952 ± 287	56	n/d	n/d	[76]
Ishizu et al. (2022)	n = 589 Female collegiate athletes	FFQ	6.1 (4.5–8.4)	n/d	487 (361–728)	n/d	4.8 (2.9–8.7)	n/d	[77]
Baranauskas et al. (2020)	n = 247 Elite athletes	24 h dietary food recall	28.8 ± 9.8 ^ 18.8 ± 7.5 ^	n/d	1227 ± 511 ^ 927 ± 474 ^	n/d	144 ± 104 ^ 88 ± 76 ^	n/d	[78]
Vermeulen et al. (2021)	n = 23 Female ice hockey players	7-day food diary	17 ± 7	52	1022 ± 256	78	4.5 ± 2.4	100	[79]
Książek et al. (2020)	n = 26 Male football players	7-day food diary	14.9 ± 2.5 # 16.7 ± 3.3 #	n/d	1179.9 ± 265.8 # 1291.5 ± 318.2 #	n/d	4.9 # 56.5 #	n/d	[80]
Gogojewicz et al. (2020)	n = 62 CrossFit athletes	3-day food diary	16.5 ± 3.6 ^ 12.6 ± 3.2 ^	n/d	1214 ± 550 ^ 894 ± 431 ^	n/d	6.3 ± 5.8 ^ 5.8 ± 6.2 ^	n/d	[81]
Dobrowolski and Włodarek (2019)	n = 41 Female soccer players	3-day food diary	8.8	n/d	646 ± 290	82.9	1.69	n/d	[38]
Jenner et al. (2019)	n = 23 Female football players	3-day food diary	12.1 ± 3.5	87	852.0 ± 288.0	61	n/d	n/d	[82]
Kim et al. (2019)	n = 36 Adolescent distance runners	3-day food diary	16.9 ± 4.4	2	582.2 ± 182.7	31	6.2 ± 3.3	n/d	[83]
Gomez-Hixson et al. (2022)	n = 75 NCAA Division III soccer players	3-day food diary	18.6 ± 6.9	n/d	1000.1 ± 402.8	n/d	4.1 ± 3.6	n/d	[84]
Masoga et al. (2019)	n = 51 Amateur bodybuilding athletes	24 h dietary food recall	173.3 ± 4.1 ^ 9.9 ± 2.6 ^	9.8	580.1 ± 355.3 ^ 477.6 ± 146.1 ^	86.3	n/d	n/d	[85]

% Below—percentage of participants with intakes below the recommended values. * Team 1 and Team 2. ^ Male and female. # Excluding and including supplementation.

## 6. Protein and Amino Acids

Protein in sports has long been of interest to many researchers as well as sports practitioners. The optimal protein supply, amino acid composition, and timing of its administration depending on training are still the subject of many investigations and recommendations of sports medicine scientific societies. Adequate protein supply is crucial for many important factors from the point of view of sports nutrition, such as post-workout recovery, muscle protein synthesis, stimulation of muscle growth, or optimization of body composition. Therefore, good-quality protein is important for maximizing athletes’ performance. As the International Society of Sports Nutrition (ISSN) pointed out in its position paper [86], athletes should consider focusing on whole food sources of protein that contain all of the essential amino acids (EAAs), because proteins that contain high proportions of EAAs and adequate leucine are most effective in stimulating muscle protein synthesis.

There are few studies indicating differences in amino acid composition between organic and conventional produce. Of those few, Armesto et al.’s (2020) study, which is an analysis of the content of selected compounds in butternut squash, showed a higher content of amino acids, including leucine, isoleucine, and valine (BCAAs), and serine, glycine, proline, threonine, phenylalanine, and a higher amount of EAAs, as well as a better ratio of essential to endogenous amino acids in organically grown squash, compared to that grown by conventional methods [23]. Also, the amino acid content of wheat grain appeared to be more health-promoting in the organic variety, but the EAA index did not differ significantly over the year between the organic and conventional crops [87]. Analysis of the composition of potato powder obtained by the organic method also showed a better amino acid composition through a significantly higher EAA content, compared to potato powder from vegetables grown by the conventional method [88]. Chitra (2013) highlighted that overall, among the organically grown crops, the quality of amino acids was higher, indicating that more essential amino acids were available [89]. The higher EAA content found in some organic plant products appears to be beneficial for muscle protein synthesis in athletes, especially vegans. However, the consensus on the overall amino acid composition of organic versus conventional foods is still unclear, and further long-term studies are needed to confirm these findings [90]. There is a relationship between the amount of EAA consumed, the postprandial concentration of EAA in the circulation, and muscle protein synthesis (MPS) [91].

The amount of protein consumed is significant in achieving the best possible training results for athletes. Admittedly, protein requirements are an individual matter, depending on a number of factors, such as body mass and composition, the sport being practiced, the training cycle, the intensity of training, or even the type of exercise. This makes it impossible to recommend specific amounts of protein for athletes in general, and recommendations are usually given in the form of ranges that an athlete should follow. However, it is worth noting that the nutritional needs of athletes indicate a higher protein requirement than those of people with average physical activity, which is mainly due to the increased oxidation of amino acids during exercise [1,3,86,92].

As one study indicated, the total protein and casein content was higher in organic milk, compared to conventional milk [63]. However, these relationships were not statistically significant. Similarly, another study found that eggs from organic hens contained significantly more protein than eggs from conventional hens [93]. Interestingly, organic eggs were significantly lighter than those of conventional eggs. A lighter product weight and higher protein content means a higher nutritional value of the product, indicating a higher utility of organic eggs. Also, soybeans, which are one of the better sources of plant protein with high nutritional value, similar in amino acid composition to animal protein, contained more of this nutrient when grown using organic standards vs. conventional methods [94]. A systematic review and meta-analysis of studies on the protein content of milk showed that there are studies presenting results in favor of both organic and conventional products. In the end, the authors of the analysis concluded that there were no significant differences as to the content of this nutrient in conventional and organic milk [11]. However, it was indicated that fat and protein content could be 20% lower in cows from organic herds. In contrast, studies of beef showed comparable protein contents in both organic and conventional meat, but with significantly higher collagen content in the former [95].

However, it should be remembered that the amino acid composition has a key role in choosing products due to protein rather than total protein amounts. The need to cover the high energy expenditure of athletes requires an increase in the amount of food consumed, and this generates a greater supply of protein. The result is that the protein requirements are usually covered with the consumption of a properly balanced ration, and often the recommended amounts are exceeded [36,96,97,98,99,100,101,102,103], but studies in which athletes did not consume adequate amounts of protein are also observed in the literature [104].

## 7. Fatty Acids

In fulfilling the nutritional needs of athletes, in addition to protein and carbohydrates, fats also play an important role. They are not only an energy component, but also a source of essential fatty acids, and fat-soluble vitamins. Intake of fat by athletes should be in accordance with public health guidelines and should be individualized based on the training level and body composition goals, while the supply of this nutrient in an athlete’s diet should not be less than 20% of the energy intake, because such restriction is likely to reduce the intake of a variety of nutrients, such as fat-soluble vitamins and essential fatty acids [1].

While adequate fat intake should, therefore, not be a problem, since athletes should consume amounts close to those recommended for the general population, it seems important to ensure an adequate supply of specific fatty acids that can affect physical performance. It has been suggested that an increased supply of *n*-3 polyunsaturated fatty acids (PUFAs) may affect post-exercise muscle recovery, injury prevention, energy metabolism, or muscle adaptation [105]. As studies indicate, dietary intake of *n*-3 PUFAs and improving the *n*-3/*n*-6 PUFAs ratio can modulate the immune and anti-inflammatory responses, which is important for an athlete’s physical performance [106]. Studies have also shown increased testosterone synthesis after the supply of *n*-3 PUFAs together with CLA (conjugated linoleic acid) [107], although studies on the use of CLA in the diet of athletes show rather limited effectiveness of the preparations in improving specific parameters, including body composition [108]. However, animal studies indicate its health-promoting properties—anticancer, anti-atherosclerotic, antidiabetic, and reducing fat synthesis effects [109].

Organic foods differ in their fatty acid profile from conventional foods. A meta-analysis conducted by Średnicka-Tober et al. (2016) showed an advantage of organic meats over conventional meats in the content of polyunsaturated fatty acids and CLA [12]. Other studies comparing conventional and organic meats also showed a higher content of polyunsaturated fatty acids and CLA in organic meats. It is worth noting that the higher content is mainly in terms of *n*-3 PUFAs, while the comparison of meats in terms of *n*-6 PUFAs showed no statistically significant differences. However, higher contents of saturated fatty acids and lower contents of monounsaturated fatty acids were also shown [53]. Another study by Ribas-Agusti (2019) has some overlap with the above observations—it showed a higher content of *n*-6 polyunsaturated fatty acids in conventional meat, with higher *α*-linolenic acid content in organic meat. However, no statistically significant differences were shown between conventional and organic meats when comparing the total content of polyunsaturated fatty acids [95]. Organically raised deer meat also had a higher content of selected fatty acids, such as CLA and DHA (docosahexaenoic acid), as well as a higher content of polyunsaturated fatty acids, including *n*-3 PUFAs, and a more optimal *n*-3/*n*-6 PUFAs ratio, compared to conventionally raised deer meat [110]. A meta-analysis comparing the composition of conventional and organic milk showed higher levels of total PUFAs, *n*-3 PUFAs, and CLA in milk from organic farms [11]. Differences in the fatty acid contents and profiles of organic and conventional meats can probably depend on the farming system. Depending on the climate, cows are grazed on grassland and pasture from April/May to September/October. The consumption of large amounts of fresh herbs—grasses and forbs—generates the synthesis of unsaturated fatty acids in the cow’s rumen, including largely CLA.

Although not all study findings are conclusive, a prevailing number of studies showed that organic products of animal origin have a higher content of *n*-3 PUFAs than those obtained by conventional methods. This is significant because in the modern diet, there is a significant shortage of *n*-3 PUFAs and an under-consumption relative to *n*-6 PUFAs. It is estimated that the mutual ratio of *n*-6/*n*-3 PUFAs is now 20–50/1. This leads to the production of more lipid mediators derived from linoleic and arachidonic acid, becoming the cause of an increased risk of atherosclerosis, the occurrence of allergic and inflammatory diseases, and other disorders. Therefore, to reduce all the health risks of excessive *n*-6 PUFAs, it is advisable to aim for a ratio of *n*-6 to *n*-3 PUFAs of 4–5/1 [111]. Thus, the consumption of organic products by athletes may improve the ratio of *n*-6 to *n*-3 in the diet, which may be important for the health and performance of athletes. However, not all studies agree, and evidence for plant products is still limited, as is the data on the bioavailability of organic products. This should, therefore, be treated with caution.

## 8. Caffeine in Organic Products

Ergogenic aids are all treatments that can improve exercise performance capacity or enhance training adaptations [108]. This, of course, includes food interventions, including diet, or the intake of specific ingredients. Any nutritional intervention that aims to improve physical performance and training is ergogenic aid, including an increased supply of specific nutrients and dietary supplementation. There are several agents that can affect an athlete’s performance, by regulating body weight or increasing performance. One such ingredient is caffeine. The content of other ergogenic agents in organic products that have a proven supportive effect on exercise has not received as much attention. This is probably due to the need for a large intake of substances to produce a specific physiological effect, and such doses are mainly available in dietary supplements designed for people with increased physical activity.

Caffeine in sports is a well-known and widely used ergogenic agent. Numerous studies have demonstrated its high effectiveness. According to the International Society of Sports Nutrition, caffeine affects muscular endurance, movement velocity, and muscular strength, sprinting, jumping, and throwing performance, as well as a wide range of aerobic and anaerobic sport-specific actions, and its effectiveness in supporting exercise has been confirmed in both untrained and high-performance athletes [112]. A recent meta-analysis found that acute caffeine intake had a small but statistically significant effect on increasing performance in team athletes [113]. Also, a recent study involving cyclists showed that caffeine intake in the form of coffee increased the rate of glycogen resynthesis within 4 h after intense exercise [114].

When comparing the composition of organic and conventional products, several works have also focused on their caffeine content. Carvalho et al. (2011) compared the caffeine content of organic and conventional coffee, showing that organic coffee contained significantly more of this alkaloid [115]. Hallmann et al. (2010) also showed higher caffeine content in organic coffees compared to coffees from conventional plantations, but this correlation applied only to instant coffees. No statistically significant differences were observed between ground coffees [116]. Interesting observations were shown in a study by Król et al. (2020), where, although conventional coffee contained significantly more caffeine than organic coffee (5.26 vs. 4.61 mg/g of product, respectively), a significant increase in caffeine content was observed during the 12-month storage period in organic coffee. These differences were not observed in coffee from conventional plantations [21]. This suggests a higher persistence and better quality of caffeine in organic products. A study by Kim et al. (2015) of green tea also found a tendency toward higher caffeine content in teas from organic vs. conventional plantations. However, the differences were not statistically significant [117]. The opposite results, however, were observed by Kazimierczak et al. (2015), where a study of green tea showed higher caffeine content in conventional products, while organic tea contained more flavonoids, tannins, and catechins [118]. In a recent review, Piyasena and Hettiarachchi (2023) pointed out that although studies indicated a higher content of polyphenols, proline, and γ-aminobutyric acid in organic compared to conventional tea, there were no statistically significant differences in caffeine content between teas under different cultivation methods [119].

One study found that the content of carnitine and carnosine in organic and conventional beef did not differ statistically significantly, although organic meat contained slightly more carnosine. It was noted, however, that organic meat contained more taurine compared to conventional meat [95].

Available research indicates that the content of ergogenic ingredients in organic and conventional products, while varying for some of them rather than others, is not large enough to expect a significant effect on athletes’ physical performance. Ergogenic agents are mostly taken in the form of supplements. One can speculate, however, that in sports in which even minimal differences in performance become important, additional factors with even a small effect on physical performance will gain significance. However, there are no studies comparing the properties and effects of caffeine from organic and non-organic coffee in terms of athlete performance.

## 9. Pesticide Content in Food and the Diet of Athletes

Data on the effects of pesticide poisoning in the respiratory tract and from consumption of food on exercise adaptation and athletic performance are fairly limited. There are, however, several studies on this subject. Coelho et al. (2024) concluded, on the basis of their study of Brazilian farmers, that chronic exposure to pesticides and coexisting fatigue can negatively affect physical fitness [120]. Interesting data were provided by Erkudov et al. (2023), who studied the physical fitness of young men living in an environmental disaster area (concerning pesticide use) in the Aral Sea region. Analysis of the data showed significantly lower values for all parameters of the physical fitness test in young men from the contaminated region compared to a relatively uncontaminated region. The authors concluded that the decline in muscle strength and endurance in young men may be influenced by organochlorine pesticides, inhibiting hemopoiesis and causing a subsequent reduction in blood oxygen capacity [121]. Fuhrimann et al. (2022), studying smallholder farmers in Uganda, showed an increased risk of sleep problems among these farmers in a pesticide-exposure-dependent manner. These problems translated into notorious fatigue and physical performance [122]. The cited studies—although still relatively few in number—clearly show the negative effects of pesticide exposure on physical fitness and thus on the ability to perform well in sport.

Pesticides have negative effects on human health, limiting the function of selected systems and organs and, in the case of excessive accumulation and lack of appropriate treatment, leading to death [123]. Chronic exposure to pesticides and their accumulation in the body can lead to a number of dysfunctions in the body, causing impaired memory and concentration, confusion, depressive states, irritability, or increased reaction times [124]. According to studies, dietary exposure to pesticides can, among other things, disrupt the endocrine system [125,126] and affect inflammation, oxidative stress, and lipid metabolism [127,128]. While the risk of pesticide poisoning from food sources is highly unlikely, it is worth noting the negative effects, such as increased oxidative stress, which athletes are particularly burdened with anyway, which can translate into physical performance.

In a literature review, Smith-Spangler et al. (2012), summarizing numerous studies comparing pesticide content in conventional and organic foods, came to the conclusion that organic foods are decidedly less contaminated, compared to conventional foods [129]. An extensive meta-analysis, based on 66 publications from around the world, found that organic crops have 4 times fewer pesticide residues than conventional crops [10]. Also, Suciu et al. (2019) noted the lower risk of pesticide contamination of organic food, compared to conventionally produced food [130]. In their study, Baudry et al. (2018) showed that people with a higher proportion of organic consumption had significantly lower urinary concentrations of pesticide metabolites compared to the group consuming conventional foods [131]. These findings were confirmed by Curl et al. (2019) in a 24-week trial involving pregnant women, where they showed that consumption of organic food was associated with lower urinary pesticide content, compared to conventional food. Moreover, as they pointed out, even a small addition of organic food, rather than switching to a 100% organic diet, showed a significant reducing effect on urinary pesticide content [132]. Another study found 91% less pesticide residues in the urine of adults after a two-week internment with an organic Mediterranean diet, compared to those on a conventional Mediterranean diet [133].

The studies described above to date clearly indicate that ingested pesticide exposure can negatively affect physical performance and exercise capacity. On the other hand, consumption of organic products significantly reduces exposure to pesticides from food intake. However, traces of these pollutants may be found due to the proximity of conventional crops. Nevertheless, it should be emphasized that organic food contains significantly lower levels of pesticides, as highlighted in the previous paragraph. All this can contribute positively to a better condition for athletes. Based on the many data on the negative effects of pesticides on human health, as well as their impact on physical activity, limiting their intake in the diets of athletes, nevertheless, seems desired.

## 10. Limitations

Despite the evidence cited above on the potential benefits of using organic products in athletes’ diets, some limitations should be pointed out. Firstly, field studies do not always show significant differences in the content of polyphenols or other bioactive compounds in organic compared to conventional crops. Many factors influence the content of bioactive compounds in plants. These are primarily the dose and type of fertilizers and the amount and type of plant protection products used. Crop rotation and the environmental factors are also important. Consequently, there is a combination of factors that together act on the metabolism and growth of the plant. Second, the number of studies on different foods is still limited. Many product groups are still overlooked, and there are no clear reports indicating the differences between some organic and conventional product versions. Because lots of products have not yet been researched enough, higher nutritional organic vs. conventional food quality is unpredictable for all kinds of food products, and there is no convincing evidence to date to conclude that conventional foods will have better or worse antioxidant potential than organic foods. It should be noted, though, that current regulations on organic food mean that the content of pesticides in organic food will be lower than in conventionally sourced food. Third, the higher contents of certain nutrients in products does not necessarily mean their better bioavailability or utilization in the body. There is no indication that ingredients from organic foods are better or worse than those from conventional foods, but more research is needed to clarify these uncertainties. Finally, more research, including intervention studies, is needed to help determine what effect organic foods have on the body, on body composition, and on physical performance and adaptation to exercise.

## 11. Conclusions

Organic food, which is intended to benefit the environment and improve its biodiversity and safety, also has a number of features that affect its nutritional value. Studies indicate that organic food can have positive applications in nutrition caused by higher contents of antioxidants, *n*-3 PUFAs, and a positive *n*-6/*n*-3 PUFAs ratio in organic vs. conventional food products, which can increase the intake of macro- and micro-nutrients in an athlete’s diet. It can positively contribute to meeting the nutrient requirements of athletes and effectively regulate the chronic oxidative stress inherent in training. Also, caffeine, in increased amounts, further emphasizes the attractiveness of organic products. While organic fruits and vegetables appear to be a good alternative to their conventional counterparts, ambiguities in the content of calcium, vitamin D, iron, or the amount of protein make it impossible to say whether such products will prevail over conventional products. Also, larger amounts of organic coffee would need to be consumed to produce an ergogenic effect. There is still a lack of studies comparing the bioavailability and utilization of ingredients from organic foods. There is also a lack of studies assessing the effect of the presence of organic products in the diet on athletes’ performance. Further research, including nutritional interventions in a group of athletes using organic foods, is strongly needed. In conclusion, it can be said that currently, organic products are an interesting alternative to conventional products in the diets of athletes, while there is still a lack of data proving that their presence in the diet provides additional exercise-related benefits.

## Figures and Tables

**Table 1:** Differences in antioxidant content of organic and conventional products.

Study	Study Material	Similar Location and Growing Conditions between Organic and Conventional Farms	Tested Compounds	Higher Content in Conventional	Higher Content in Organic	Reference
Rachtan-Janicka et al. (2021)	Black currant	Yes	Polyphenols, vitamin C, anthocyanins	-	Vitamin C, total polyphenols, total phenolic acids, total flavonoids, anthocyanins	[22]
Hallmann et al. (2019)	Pickled bell pepper	Yes	Dry matter, carotenoids, polyphenols	Phenolic acids	Flavonoids, carotenoids	[19]
Crecente-Campo et al. (2012)	Strawberries	Yes	anthocyanins, ascorbic acid, total phenolic content	-	Anthocyanins, ascorbic acid	[29]
Król et al. (2020)	Coffee beans	Yes/n/d	Dry matter, polyphenols, caffeine, flavonoids	Kaempferol, quercetin-3-O-glucoside and flavonoids in stored coffee and quercetin-3-O-rutinoside in freshly roasted coffee	Total phenolic acids, phenolic acids, flavonoids in freshly roasted coffee	[21]
Ponder and Hallmann (2019)	Raspberries	Yes	Dry matter, phenolic acid, flavonoids, anthocyanins	-	Total polyphenols *	[20]
Armesto et al. (2020)	Butternut squash	n/d	Physical and chemical properties, minerals, vitamins, amino acids, antioxidant components	Folic acid, β-carotene	Tocopherol	[23]
Średnicka-Tober et al. (2020)	Apples	Yes	Dry matter, phenolic acids, flavonols, vitamin C	-	Phenolic acids, flavonols	[24]
Dutra et al. (2018)	Grape juices and wines	n/d	Total phenolic content, antioxidant activity, minerals	Anthocyanins	-	[27]
Skupień et al. (2011)	Raspberries	Partly/No	Dry matter, soluble solids, titratable acidity, sugars, vitamin C, total polyphenol content	-	-	[28]
Kopczyńska et al. (2020)	Courgettes	Yes	Dry matter, phenolic compounds, carotenoids, chlorophylls, vitamin C	-	Polyphenols, phenolic acids, flavonoids, carotenoids, chlorophylls	[30]
Hallmann and Rembiałkowska (2011)	Sweet bell pepper	Yes	Dry matter, vitamin C, carotenoids, polyphenols	-	Vitamin C, carotenoids, phenolic acids	[31]
Kazimierczak et al. (2013)	Beetroot and beetroot juices in LNF ^ and HNF ^ level	Yes (for both juices and beetroots)	Dry matter, sugars, acidity, vitamin C, phenolic compounds, betacyanins,	Phenolic acids (LNF for beetroots), phenolic acids (HNF for juices), flavonoids (LNF for beetroots)	Vitamin C (for both beetroot and juices)	[32]

Columns “higher content in conventional” and “higher content in organic” contain statistically relevant differences in antioxidants. * Only 1 year of production. ^ LNF—low-nitrogen fertilization; HNF—high-nitrogen fertilization.
